# Testing the Usefulness of the Evidence Integration Triangle for Implementation of EIT-4-BPSD

**DOI:** 10.1093/geroni/igab046.589

**Published:** 2021-12-17

**Authors:** Barbara Resnick

**Affiliations:** University of Maryland School of Nursing, Baltimore, Maryland, United States

## Abstract

The effectiveness of evidence-based practices, such as use of behavioral interventions, can be improved when delivered under conditions of an implementation framework. This pragmatic trial used the Evidence Integration Triangle (EIT) which is a parsimonious, community-engaged participatory framework that brings evidence and facility stakeholders together. Active engagement empowers key stakeholders to integrate evidence into practice using a simple three-pronged framework: (1) A participatory implementation process which was done via monthly meetings and weekly emails between stakeholders and a clinical expert as they worked on facility based goals; (2) Implementation of the four steps delineated in the EIT-4-BPSD; and (3) evaluation using practical progress measures. There was some evidence of implementation of the EIT-4-BPSD based on participation in Stakeholder team meetings, settings working towards goal achievement, and increased use of behavioral interventions among staff. The EIT approach is a useful implementation framework to help change staff behavior in long term care.

